# Ameloblastic Fibro-Odontoma of the Posterior Mandible: A Rare Pathological Entity

**DOI:** 10.7759/cureus.46264

**Published:** 2023-09-30

**Authors:** Mahima Goel, Ali Qamar, Mimansa Daftary, Sujata Chhabile, Shruti Pundkar

**Affiliations:** 1 Department of Oral and Maxillofacial Surgery, Pacific Dental College and Research Centre, Udaipur, IND; 2 Department of Oral and Maxillofacial Surgery, Teerthankar Mahaveer Dental College and Research Institute, Moradabad, IND; 3 Department of Public Health Dentistry, JMF's ACPM Dental College, Dhule, IND; 4 Department of Public Health Dentistry, Vidarbha Youth Welfare Society Dental College, Amravati, IND

**Keywords:** benign tumor, enucleation, posterior mandible, odontogenic tumor, ameloblastic fibro-odontoma

## Abstract

Ameloblastic fibro-odontoma (AFO) is a rare, slow-growing neoplastic lesion classified as a benign, epithelial mixed odontogenic tumor with odontogenic mesenchyme. This tumor demonstrates the histological features characteristic of both ameloblastic fibromas and complex odontomas. The clinical manifestation of AFO is typically characterized by asymptomatic enlargement of the jawbones. Radiographically, it presents as a distinct radiolucent region, indicating the presence of radiopaque substances with varying degrees of irregularities in size and morphology. Standard therapeutic intervention involves enucleation. Despite its benign nature, AFO can cause significant morbidity if left untreated. Therefore, prompt diagnosis and appropriate management are essential to ensure optimal patient outcomes. The following case report details the clinical presentation and management of an 18-year-old male with an AFO lesion located in the posterior mandible. This particular case was treated with conservative measures involving surgical enucleation.

## Introduction

Ameloblastic fibro-odontoma (AFO) is an infrequent, gradually progressing, expansile mixed odontogenic neoplasm [1]. The aforementioned lesion exhibited histological characteristics and biological features similar to those of an ameloblastic fibroma. However, in AFO, one or more odontogenic epithelial cellular foci persist during the differentiation process, leading to the production of both enamel and dentin [2]. According to the 2017 classification of the World Health Organization (WHO), AFO should now be classified as an odontoma rather than an odontogenic tumor [3]. It typically manifests in younger individuals and displays a mildly significant tendency toward males. It has been observed to occur with equal frequency in both the maxilla and mandible, and its commonly observed site of occurrence is in the region posterior of the premolars and molars [4]. AFO, which frequently affects young children, is often challenging to diagnose before surgical intervention due to the lack of pre-surgical pathological information [1]. This is typically resolved by relying on observations obtained from a presurgical biopsy performed under local anesthesia. Consequently, clinical diagnosis assumes an exceedingly significant role in the preemptive delineation of surgical procedures, particularly with regard to determining the feasibility of conserving teeth situated in close proximity to the lesion [5]. The purpose of this paper is to report a clinical case of ameloblastic fibroodontoma (AFO) in an 18-year-old individual with a lesion involving the posterior mandible that was managed through surgical enucleation.

## Case presentation

An 18-year-old adolescent male was referred to the Department of Oral and Maxillofacial Surgery in March 2020 with a primary concern of swelling on the right side of his face for one year. The patient’s history indicated the existence of an indistinct, asymptomatic, and slowly developing bulge over a span of one year. The medical history of the patient was noncontributory. The patient did not report any history of weight loss, alcohol or tobacco usage, fever, or past medications for the swelling. The patient was referred by the local dentist to the Department of Oral and Maxillofacial Surgery. Moreover, a discernible slight asymmetry of the face was noted on the right side and linked to engagement of the posterior area of the mandibular bone. The medical records did not have any significant findings, and the dental history did not indicate any evidence of local injury or infectious activity at the site of the lesion. On extraoral examination, it was determined that the enlargement exhibited an osseous morphology characterized by a rigid nature, with the integumentary covering displaying typical characteristics. No paresthesia was identified in the affected area, although displacement of the inferior alveolar nerve was detected. During intraoral examination, an asymptomatic expansion was observed on the posterior aspect of the right mandibular side. The mucosal layer exhibited unremarkable pigmentation, which was consistent with the overlying skin. The swelling spanned from the second to third molar regions and displayed buccal and lingual cortical expansion.

**Figure 1:**
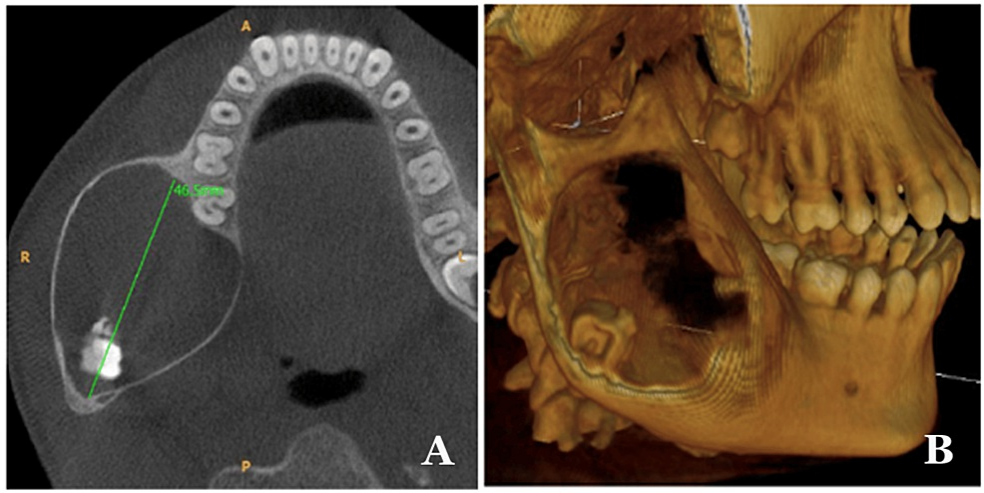
Computed tomography of the mandible showed 38.3 mm of mediolateral expansion of the cortical plates. [B] 46.5mm anteroposterior expansion of the lesion showing intact borders of the mandible

Cone-beam computed tomography (CBCT) was used to identify a predominantly hypodense area of unilocular nature. This area involved the right body, angle, and ramus of the mandible, with a thin corticated margin. The area extended anteriorly from the lower right first molar region to the posterior border of the ramus. The dimensions of this area were 46.5 mm anteroposteriorly, 38.3 mm mediolaterally, and 53.8 mm superioinferiorly, as shown in Figure 1A. A displaced third molar in the right mandibular region was observed in association with calcification flecks near the angle of the mandible (Figure 1B). The presence of a bony crypt related to the third molar was not observed. However, both the lingual and buccal cortical plates appeared to be attenuated and displayed an obvious expansion without any discernible disruption of continuity.

**Figure 2:**
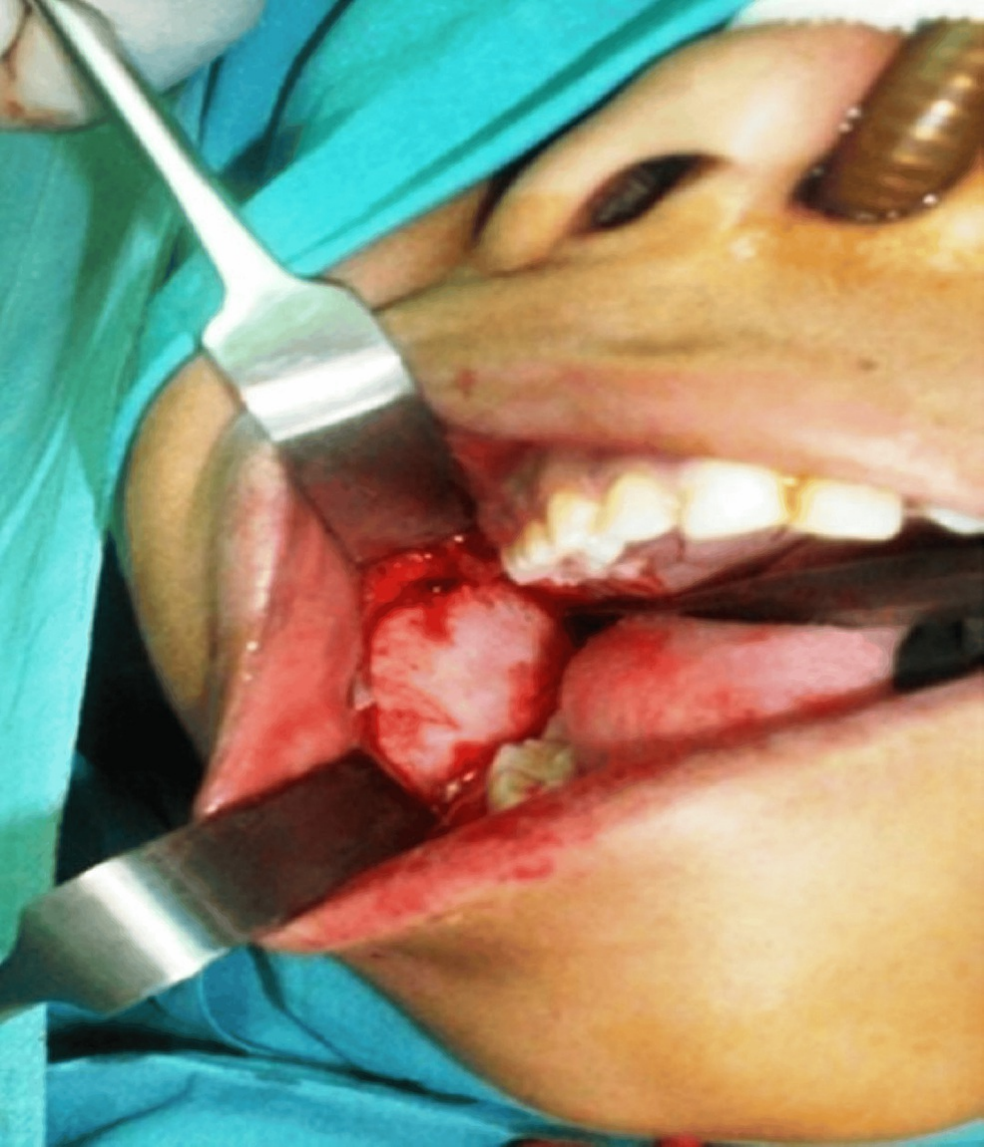
Intraoral approach showing surgical enucleation of the lesion from the right posterior mandible

Based on a comprehensive clinical and radiographic assessment, a provisional diagnosis of calcifying cystic odontogenic tumor was given, including the differential diagnosis of odontoameloblastoma, complex or compound odontoma, ameloblastic fibroma, or ameloblastic fibro-dentinoma. Routine hematological investigations were found to be within the normal limits. Following a scrupulous presurgical evaluation, the procedure was performed under general anesthesia, with an intraoral approach selected for enucleation of the lesion along with the third molar. Extraction of the right second mandibular molar was performed because of root resorption. Curettage of the bony walls was subsequently performed, as shown in Figure 2. Bone smoothening was accomplished, and the excision site was securely sutured with 3-0 vicryl. Intermaxillary fixation was performed to immobilize the mandible and prevent any pathological fracture of the mandibular body.

**Figure 3:**
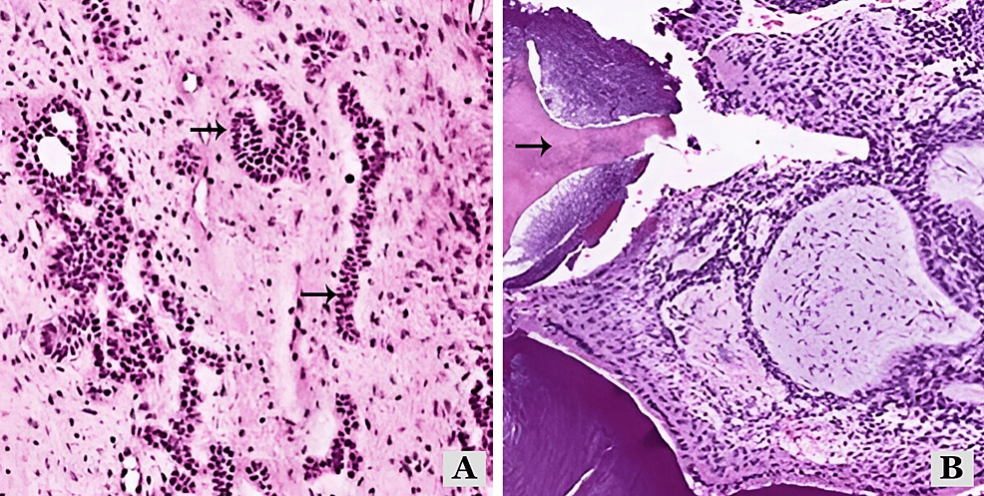
Histopathology showed [A] islands and cords of ameloblast-like cells at 40 X magnification [B] calcified mass in the hypocellular stroma at 40 X magnification

The lesional tissue obtained underwent histopathological examination in the Department of Oral Pathology. Microscopic examination of sections stained with hematoxylin and eosin revealed islands, cords, and strands of ameloblast-like cells in a loose, immature connective tissue similar to dental papillae. Calcified flecks were also observed in the stroma near the aforementioned islands. Stromal calcifications indicate the possibility of dentinoid or enameloid material (Figure 3).

**Figure 4:**
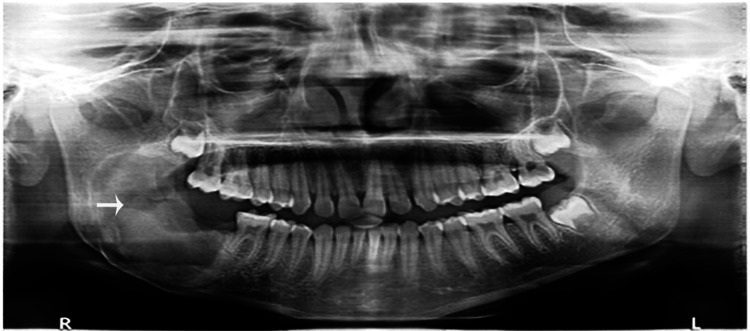
Successful healing of the lesion site (arrowhead) on a two-year follow-up visit

The final diagnosis of the tumor was ameloblastic fibro-odontoma. The soft tissue healing process was uneventful, with no evidence of recurrence during the two-year follow-up period (Figure 4).

## Discussion

Ameloblastic fibro-odontoma, popularly known as AFO, is a pathological condition that can be categorized as a slowly developing benign, mixed odontogenic neoplasm [1]. This type of tumor is predominantly observed in the jaw bones, with a low incidence rate of approximately 1%-3% [4]. The available literature substantiates that AFO predominantly targets juveniles, who have a mean age of 9.4±3.5 years, and evinces a predilection toward males, indicating an M/F ratio of 2.3:1 [6]. Furthermore, it is notable that the condition primarily affects the mandible, which differs from the conclusions presented in the 2005 WHO classification. The assertion made by the latter posited the absence of sex predilection in the distribution of the lesion, which was equally distributed across the mandible and maxilla, particularly in the molar region [6]. Notably, several instances of the lesion were correlated with an unerupted tooth situated in the periphery, with the corresponding tooth displaced in the apical direction. This observation suggests that the origin of the lesion is from the remnants of the odontogenic epithelium located above the impacted tooth [7].

According to the 2017 WHO classification, AFO has been designated as a precursor of complex odontoma [3]. However, Soluk-Tekkesin et al. analyzed a series of cases of AFO and odontomas and concluded that AFOs affect individuals between the ages of 3 and 16 years and possess a size greater than 2 cm [8]. In contrast, odontomas tend to affect older individuals whose age ranges around 27 years and whose size is less than 2 cm. Hence, it is imperative to consider AFO with measurements exceeding 2 cm as a true neoplasm rather than a hamartomatous odontoma [8]. Radiographic examination of the AFO revealed a distinct and identifiable radiolucency that was well-circumscribed and round-to-ovoid in shape. This radiolucency is surrounded by a thin sclerotic margin that contains various amounts of radiopaque material of irregular size and form. Notably, the ratio of radiopaque to radiolucent areas may differ from one lesion to another, indicating the uniqueness of each case [2]. This case was documented in an 18-year-old male who presented with an enlargement exceeding 5 cm in the posterior section of the mandible with flecks of calcification indicative of ameloblastic fibroodontoma.

Histopathological analysis has the capacity to offer a precise determination in instances that encompass odontoameloblastoma, complex odontomas, calcifying epithelial odontogenic tumor (CEOT), ameloblastic fibroma (AF), and ameloblastic fibrodentinoma (AFD) due to the resemblance in their clinical and radiological characteristics [1].

Odontoameloblastoma is a combination tumor of complex odontoma and ameloblastoma with an invasive nature, as seen in classical ameloblastoma. Radiographically, they represent unilocular radiolucency associated with impacted molars and radiopaque substances. They may show perforation of cortical plates, which is not a feature of AFO. Histologically, odontoameloblastomas show mature stroma with epithelial islands of ameloblastic cells and calcification [8].

There is a current and ongoing debate regarding the consideration of AF, AFO, AFD, and odontomas as distinct entities or varying stages in the developmental process of odontomas [9]. According to the 2017 WHO classification, certain lesions that exhibit histopathologic similarity to an AFO are likely indicative of the development of odontomas [3]. Furthermore, it is plausible that the histopathological appearance of an AFO may be indistinguishable from that of developing odontomas. In such instances, the evaluation of concurrent clinical and radiological features may be a valuable tool for establishing diagnostic differentiation.

Histopathologically, it is imperative to note that a calcifying epithelial odontogenic tumor (CEOT) is typified by the noticeable presence of epithelial cells, homogenous eosinophilic amyloid-like material, and calcification. Epithelial cells are organized, forming nests and sheets that are polygonal in shape and structure. In addition, the cells have clear to eosinophilic cytoplasm and vesicular nuclei with prominent nucleoli [10]. However, it is noteworthy that the aforementioned features were absent in our case.

Microscopically, AF comprises various strands and small islands of the odontogenic epithelium. However, unlike the enamel organ-like structures that are commonly found in AFO, AF does not possess such structures. Moreover, the fibrous elements in AF may range from cellular to mature collagenous tissue. Despite this, it is noteworthy that the primitive dental papilla-like appearance was not obvious in AF [11]. Furthermore, the ectomesenchymal component of AFO bears a striking resemblance to the dental papilla. 

Ameloblastic fibro-odontoma (AFO) shares some similarities with ameloblastic fibrodentinoma; however, AFO contains both enamel and dentinal materials, whereas ameloblastic fibrodentinoma solely comprises dentinal materials. Consequently, it can be noted that, upon radiological observation, the characteristics of AFO entail a greater presence of opaque and denser elements within the lesion as opposed to ameloblastic fibrodentinoma. The observable difference in radiology between AFD and AFO is distinctly evident by the multilocular nature of AFD lesions, which sets them apart from the unilocular lesions of AFO [12].

In terms of radiology and histology, unlike AFO, odontomas encompass a profoundly calcified component comprised of enamel, dentin, cementum, and connective tissue pulp. The aforementioned components are observed in the advanced stages of compound odontoma, where oral maturation attains a state of completeness [9].

In the current case report, the ameloblastic fibro-odontoma exhibited a considerable size exceeding 5 cm and was well encapsulated with minimal inclination without localized invasion. Consequently, the recommended course of treatment involves conservative surgical intervention, entailing enucleation, in conjunction with the extraction of a non-erupted tooth. However, it is worth noting that documented cases exist in which the involved teeth have been preserved [13]. Despite the lesion’s limited propensity for recurrence, there have been instances where conservative treatment aimed at preserving the associated teeth has yielded reports of recurrence [13]. Consequently, to eliminate the possibility of recurrence, the unerupted third molar was extracted in conjunction with AFO enucleation.

## Conclusions

The clinical and radiographic features of AFO are heterogeneous, and its diagnosis can be determined solely by histological examination. Some authors contend that ameloblastic and complex fibro-odontomas are sequential phases of the same pathological entity, whereas others maintain that these lesions are distinct pathologies. Although infrequent, it is important to consider the possibility of its presence in the differential diagnosis of intraoral radiolucencies that comprise radiopaque material, particularly when managing pediatric patients. Irrespective of its classification, considering its benign behavior, minimal invasiveness, and low recurrence rate, the preferred method of treatment is a conservative approach, which typically involves enucleating the lesion and extracting the associated tooth to prevent future recurrences. In this study, we observed a positive postoperative course with no signs of recurrence. Owing to the potential for malignant transformation, it is strongly advised that long-term monitoring and observation be implemented.
